# Descriptive Patterns of Ketamine Administration Among Adult Emergency Department Patients at a Single Academic Center in Saudi Arabia

**DOI:** 10.3390/jcm15135246

**Published:** 2026-07-05

**Authors:** Rana Aljadeed, Raniah Aljadeed, Nora Kalagi, Hailah Almoghirah, Salha Jokhab

**Affiliations:** Department of Clinical Pharmacy, College of Pharmacy, King Saud University, Riyadh 11451, Saudi Arabia

**Keywords:** ketamine, emergency department, procedural sedation, dose

## Abstract

**Background/Objectives**: Ketamine use in emergency departments is expanding for the management of agitation, procedural sedation, and rapid sequence intubation. We aim to describe the indications and characteristics of ketamine administration in emergency department patients in Saudi Arabia through retrospective chart review. **Methods**: This was a single-center, retrospective chart review conducted at an academic medical center. The study population included patients who received intravenous (IV) or intramuscular (IM) ketamine in the emergency department between 1 January 2023, and 31 December 2024. Patients younger than 18 years of age or those who were pregnant were excluded. **Results**: A total of 192 patients were included. The median ketamine dose was 50 mg (a total range of 20–240 mg). Procedural sedation was the primary indication (79%), followed by pain management (12.5%), rapid sequence intubation (4.7%), and agitation (2.1%). Nearly all patients (99.5%) received ketamine intravenously, and 83.9% received it in conjunction with other medications. For procedural sedation, ketofol was the most common combination therapy, accounting for 83.6% of combination cases. No procedural failures, need for additional sedatives, or redosing within an hour were reported for procedural sedation. For pain management, ketamine was predominantly administered for musculoskeletal pain (50%) and was most often used as monotherapy (58.3%). Notably, no documented adverse effects were observed post ketamine administration across all indications. **Conclusions**: Among patients who received ketamine in emergency departments in Saudi Arabia, its administration was frequent, particularly for procedural sedation and acute pain management. In this study we found no adverse events or procedural failures were documented. Further research is warranted to validate these findings.

## 1. Introduction

In the patient care field, the popularity of ketamine continues to rise [1]. The simultaneous effects of ketamine have differentiated the drug as a versatile solution for treating patients both in and out of the operating room [2]. Ketamine is an analgesic, hypnotic, and amnestic drug [3]. Its effects are largely attributed to blocking of the excitatory effects of glutamate, resulting in noncompetitive antagonism of N-methyl-D-aspartate (NMDA) receptors. It also targets α-amino-3-hydroxy-5-methyl-4-isoxazolepropionic acid (AMPA) receptors and sigma 1 receptors [2]. Another advantage that sets ketamine apart from other medications is the variety of ways in which it can be administered: oral, intravenous, intraosseous, intramuscular, and intranasal routes. This versatility enhances the value of ketamine across various patient care realms, including emergency medicine. Ketamine also has multiple indications. In emergencies, ketamine is mainly used for agitation, procedural sedation, and rapid sequence intubation (RSI) [1].

Each of these indications has a specific dosing recommendation and preferred route of administration. As the clinical uses of ketamine continue to expand, data is needed for better assessment of its safety and efficacy, particularly in the emergency department (ED) setting.

In Saudi Arabia, the published literature on ketamine use in the ED remains limited but growing. Bin Salleeh et al. conducted a retrospective cross-sectional study of 179 children in ED who underwent procedural sedation [4]. Ketamine was the predominant used agent, employed in 90% of cases, with the most common indication being procedural sedation for fracture and dislocation reduction (60.9%). On the other hand, Alghadeer et al. examined ketamine use for acute agitation over a seven-year period from June 2015 to March 2022 [5]. Only 20 patients received ketamine as the initial calming agent over this period. This highlights how infrequently it is used for this indication in EDs.

This study aimed to describe the indications, dosing patterns, routes of administration, co-administered medications, and documented outcomes of ketamine administration among adult ED patients at a single academic center in Saudi Arabia.

## 2. Methods

### 2.1. Study Design and Setting

This was a single-center, retrospective chart review conducted at an academic medical center. The study evaluated patients who received ketamine within the Emergency Department (ED) between 1 January 2023, and 31 December 2024. The study protocol was approved by the institutional review board.

### 2.2. Participants and Case Identification

Subjects included patients aged 18 years or older who received intravenous (IV) or intramuscular (IM) ketamine within the ED during the study period. Patients were excluded if they were less than 18 years of age or pregnant. Pregnancy status was identified through an electronic medical record (EMR) review of documented clinical notes. Patients who received ketamine outside the ED but during the same hospital encounter were excluded.

Ketamine cases were identified via medication administration records (MAR). While the institution primarily uses IV and IM routes, other routes such as oral, intranasal, or intraosseous were either not utilized or cases were excluded to maintain cohort homogeneity. The institution uses a standard ketamine vial concentration of 500 mg/10 mL (50 mg/mL) for all administrations.

### 2.3. Variables and Outcomes

The primary unit of analysis was the individual ketamine administration. Repeat visits by the same patient were treated as independent observations; in this cohort, four patients received ketamine over multiple ED visits. Data were extracted from the EMR by three independent reviewers using a standardized data collection form. Inter-rater reliability was performed to ensure consistency, and any ambiguous documentation was resolved through reviewer consensus.

Relevant data points included:

- Demographics: age, sex, and body weight.
- Administration details: dosage (mg and mg/kg), route, and co-administered medications (e.g., propofol, fentanyl, midazolam, and paralytics).
- Clinical context: indication for use (procedural sedation, pain management, RSI, agitation, status asthmaticus, or status epilepticus) and seizure history.

The primary outcomes included:

- Procedural success: defined by the absence of procedural failure or the need for rescue sedation.
- Procedural failure: defined as the inability to complete the intended procedure under ketamine sedation, requiring alternative sedation or abandonment of the procedure.
- Rescue sedation: defined as the administration of any additional sedative agent (e.g., propofol, midazolam) due to inadequate sedation with ketamine alone.
- Dosing requirements: incidence of redosing within one hour of the initial administration.
- Adverse events: active screening for oxygen desaturation; apnea; the requirement for airway interventions (including supplemental oxygen, bag-mask ventilation, or intubation); clinically significant hemodynamic changes (hypotension or hypertension); aspiration; unplanned escalation of care; emergence reactions; nausea and vomiting; laryngospasm; and hypersalivation.

## 3. Statistical Analysis

All data analyses were performed using SPSS (version 29, SPSS Inc., Chicago, IL, USA). The normality of the data was assessed using the Shapiro–Wilk test and visual inspection of histograms. Descriptive statistics were used to summarize the data. Continuous variables were reported as means ± standard deviation (SD) or medians with range (minimum–maximum) when the data is skewed. Categorical variables were expressed as frequencies and percentages n (%). Data were stratified by clinical indication for comparative analysis. No formal inferential statistical comparison or hypothesis testing were planned because of the descriptive nature of the study. Accordingly, *p*-values are not reported. Missing data were minimal and handled by complete-case analysis, no amputation was applied. The denominator for each variable reflects the number of patients with available data. For the safety outcome, the incidence of adverse events was reported with exact 95% confidence intervals calculated using the Clopper–Pearson method.

## 4. Results

A total cohort of 192 patients were included in the study. The mean age was 37 years (with a total range of 18–81 years). The majority were male (68.2%), and the mean body weight was 72.4 kg. The median ketamine dose administered was 50 mg (total range 20–240 mg). Four patients received ketamine over multiple ED visits. Nearly all patients (99.5%) received ketamine via the intravenous route, and 83.9% were treated with ketamine in conjunction with other medications (Table 1). One standard ketamine vial concentration was used at our institution (500 mg/10 mL), which was used for all ketamine doses.

### 4.1. Indications for Ketamine Administration

**Figure 1:**
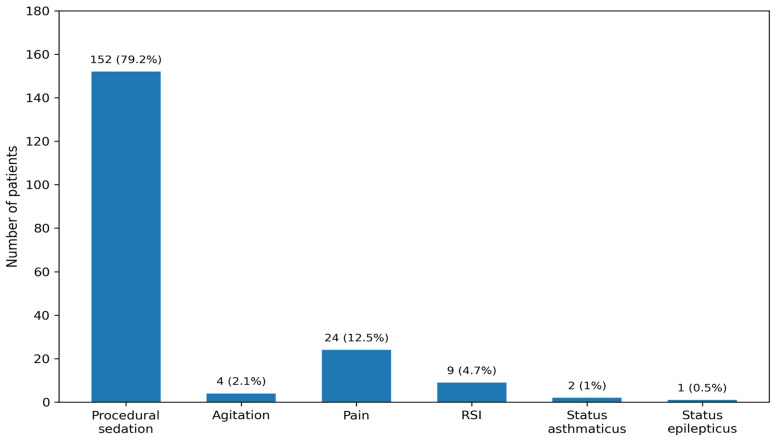
Distribution of ketamine use by indication (n = 192).

The main indication was procedural sedation, which accounted for over 79% of patients. Other indications included pain management (12.5% of patients), rapid sequence intubation a.k.a. RSI (4.7% of patients), and agitation (2.1% of patients). Less common indications were status asthmaticus (observed in 1% of patients) and status epilepticus (observed in one patient). Data were analyzed based on the indication for ketamine as follows (Figure 1).

#### 4.1.1. Ketamine Use in Procedural Sedation

The majority of patients were male (69%) with a mean age of 37 years (total range 18–77 years). Ketamine was administered intravenously to all participants who underwent procedural sedation. The mean dose of ketamine was 55.6 mg (0.79 mg/kg), with a range of 0.13–2.82 mg/kg.

**Figure 2:**
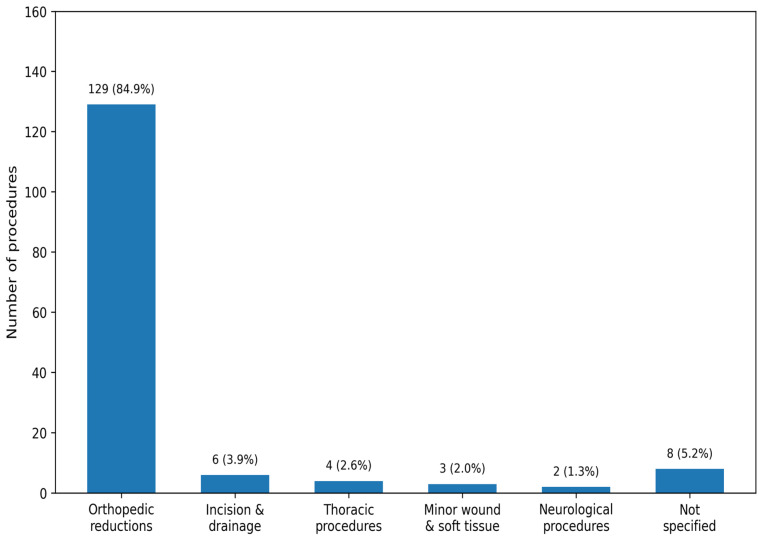
Distribution of ketamine use for procedural sedation (n = 152).

Procedures were categorized as shown in Figure 2. Nearly 90% of patients received ketamine in combination with other medications. Among the combination therapies, propofol was the most common co-administered drug (83.6%), followed by fentanyl (3.3%) and midazolam (1.3%). The dosing information (mg/kg) for ketamine is provided in Table 2. The ratio of ketamine to propofol was 1:1 in 53.5% of patient cases. At our institution, ketofol was administered as separate sequential injections of ketamine and propofol rather than as a premixed syringe, consistent with local emergency department practice. There were no reported incidents of procedural failures, incidents in which patients required other sedative agents, or redosing of ketamine within the hour following initial ketamine administration. Details about dosing information (mg/kg) for ketofol are provided in Table 3.

#### 4.1.2. Ketamine Use in Pain Management

The majority of patients were male (71%), with a mean age of 36.9 years (over a range of 20–75 years). Ketamine was primarily utilized in treating musculoskeletal pain (50% of cases). It was also used to treat sickle cell crises (12.5% of cases) and visceral pain (8% of cases). Ketamine was administered intravenously to all patients, with a mean dose of 29 mg (0.43 mg/kg) over a range from 0.15 to 1.40 mg/kg. Eighteen (75%) patients received subanesthetic doses of ≤0.5 mg/kg, with nine patients receiving 0.1–0.3 mg/kg and nine patients receiving 0.31–0.5 mg/kg. The remaining six patients (25%) received doses of >0.5 mg/kg. The majority of patients received ketamine as monotherapy (58.3%), while the remainder received combination therapy (41.7%). When combined, morphine was the most frequent co-administered analgesic (used in 20.8% of cases), followed by fentanyl (16.7%) and propofol (4.2%).

#### 4.1.3. Ketamine Use in Rapid Sequence Intubation (RSI)

The majority of patients were male (67%) with a mean age of 65 years (over a range from 35 to 81). Ketamine was administered intravenously to all participants. The mean dose was 102 mg (1.62 mg/kg, over a 0.91–4 mg/kg range). For all patients using this indication, ketamine was used in combination with paralytics. Rocuronium was the most common co-administered drug, used in 55.6% of cases, followed by succinylcholine in (33.3% of cases) and fentanyl (11.1% of cases). There were no reports of patients requiring other induction agents or patients requiring redosing of ketamine within an hour of initial ketamine administration.

#### 4.1.4. Other Ketamine Indications

The remainder of patients received ketamine for other indications not mentioned above. This included agitation, for which patients received a mean ketamine dose of 75 mg (1.27 mg/kg, over a range from 0.46–2.41 mg/kg). In terms of the route of ketamine administration, three patients received ketamine intravenously and one received it intramuscularly. All patients received ketamine as part of a combination therapy, with other medications including haloperidol and/or benzodiazepines. No patients required any other sedation within an hour of ketamine administration, and no patients required redosing of ketamine. For other indications—status asthmaticus and status epilepticus—ketamine was utilized as monotherapy and in combination. It was administered intravenously for all patients.

### 4.2. Adverse Effects

No documented adverse events were observed among the 192 patients who received ketamine, corresponding to an observed incidence of 0.0% (95% exact confidence interval [CI], 0.0–1.9%).

## 5. Discussion

Literature describing ketamine use in the ED is limited, leading to controversy over its efficacy and significant variations in clinical practice. This single-center, retrospective chart review provides insights into ketamine utilization within a large, tertiary-care, academic ED. During our observation period, ketamine in any form was administered to 192 patients for 6 different indications, demonstrating its wide utilization and versatility within the emergency realm. It was frequently utilized for traditional indications such as procedural sedation and pain. It was used infrequently for the management of these indications: agitation (2.1%), RSI (4.7%), status asthmaticus (1%), and status epilepticus (0.5%). This pattern of indication distribution is broadly consistent with findings from comparable contemporary descriptive reviews conducted in large academic emergency departments where procedural sedation and agitation similarly predominated [1]. Notably, we observed no reports of procedural sedation failure nor of patients requiring additional sedative agents or redosing of ketamine up to 30–60 min following its administration.

While agents such as propofol, fentanyl, and midazolam are commonly used for procedural sedation, the use of ketamine for this purpose has grown considerably in recent years [2,6]. Current emergency medicine guidelines support the use of ketamine as a safe and appropriate agent for procedural sedation in the emergency department [7,8]. Largely, this is due to the unique pharmacological profile of ketamine, which provides potent analgesic and sedative effects, elicits rapid onset of sedation, and demonstrates short duration of action [9,10]. Additionally, because ketamine preserves respiratory and cardiovascular function, it may be preferable over other agents such as propofol in patients where hypotension or respiratory depression is a concern. Despite these benefits, ketamine has several undesirable side effects including nausea, vomiting, and emergence phenomena, which may occur at doses as low as 1 mg/kg. To mitigate these effects, the co-administration of ketamine with propofol, referred to as “ketofol,” has gained interest in the emergency realm [9]. This combination preserves the sedative benefit of ketamine while reducing its side effects, owing to the lower doses of each drug used.

The findings of the present study reflect this growing trend, as we found that ketofol was the most commonly administered combination therapy for procedural sedation, representing 83.6% of cases of ketamine combination therapy. Compared with propofol, ketofol was associated with fewer respiratory adverse events [11,12]. However, evidence regarding overall sedation quality was mixed: two of five included studies reported better sedation quality with ketofol, whereas the remaining three found no significant difference. Compared with ketamine monotherapy, ketofol appears to offer a more consistent safety advantage, with studies conducted specifically in the ED setting concluding that ketofol is both safe and more effective than ketamine alone, with improved recovery profiles and reduced emesis [13,14]. A 2024 systematic review and meta-analysis demonstrated that ketofol was favored by clinicians due to its shorter recovery time and lower incidence of nausea and vomiting [15]. In sum, ketofol’s principal benefit lies in mitigating the adverse effects of its individual components rather than achieving absolute superiority, a conclusion consistent with this study’s own caveat that the observed utilization patterns should not be interpreted as evidence of superiority. Importantly, the present study was not designed to evaluate the comparative safety or efficacy of ketamine or ketofol relative to propofol, opioids, or other sedative and analgesic agents. Therefore, the utilization patterns observed in this cohort should not be interpreted as evidence supporting the superiority of ketofol over alternative regimens. References to prior studies are provided solely to contextualize the local practice patterns identified in this cohort and should not be construed as confirmation of findings reported in previous comparative investigations.

Pain management was the second most common indication for ketamine utilization in this study, representing 12.5% of all cases of ketamine administration. This highlights its growing role as a non-opioid analgesic in the ED. Sub-dissociative dose ketamine (SDK), typically administered at 0.1 to 0.3 mg/kg, has been shown to be an effective and safe alternative for treating acute pain, particularly in opioid-tolerant patients or when seeking to minimize opioid-related side effects like respiratory depression and sedation [16]. In meta-analyses and randomized controlled trials, SDK has been found to be non-inferior to fentanyl and morphine in managing acute pain among children and adults presenting to the emergency department [17,18,19]. Few studies demonstrated that SDK provides analgesia comparable to that of opioids for acute pain in the ED, with a distinct and generally mild side effect profile [20,21,22]. Research examining ketamine prescribing practices has demonstrated that adherence to indication-specific dosing ranges improves significantly following implementation of standardized order panels and provider education, suggesting that practice variation in the absence of formal institutional protocols is common [23]. The frequency of SDK use observed in this study, particularly for musculoskeletal pain, suggests that SDK was used with some regularity within this institution. These observations provide a local descriptive reference point and generate specific hypotheses for future prospective investigation within the Saudi ED context.

The zero adverse event rate documented in the present study is notably lower than rates reported in the contemporary literature and warrants careful interpretation. In one systematic review and meta-analysis of 50 randomized controlled trials comprising 5,398 patients, although ketamine was expected to minimize respiratory events, findings were mixed [24]. Ketamine and its combinations produced low incidences of hypoxia and apnea in some cases, whereas propofol–ketamine combinations exceeded the pooled effect size. Additionally, a comparable single-center retrospective cohort study reported that 5% of patients had a documented adverse event, including one incidence of ketamine-induced laryngospasm requiring intubation [1]. The absence of any documented adverse events in the present study falls well below these benchmarks and should not be interpreted as evidence of zero risk. Rather, the zero adverse event rate should be interpreted with caution, as it may reflect under-detection due to documentation limitations rather than a true absence of harm.

This study is limited by its single-center, retrospective study design. As such, our findings are limited in their generalizability. The retrospective nature of the study relies on medical record documentation, introducing the potential for variability in data completeness and accuracy, particularly with regard to ketamine administration within the often-chaotic emergency department. Accordingly, the utilization patterns, dosing practices, and clinical outcomes described in this cohort may not be representative of ketamine use in other emergency departments within Saudi Arabia or internationally. These findings should therefore be interpreted with caution when extrapolating them to broader clinical or policy settings. Also, our findings describe the indications and characteristics of ketamine use among those who received it and do not represent overall utilization rates or the prevalence of its use within the broader patient population. Future prospective studies incorporating comprehensive and appropriate comparison groups are needed to provide a more complete understanding of ketamine utilization trends and factors influencing prescribing practices in Saudi Arabia. Ketamine dosing extraction was based on electronic medical record documentation of patient weight, which is complicated by the practice in the ED of estimating patient weight, leading to a potential divergence between intended and actual dosing that warrants consideration when assessing observed outcomes, as the intended and actual mg/kg dosage may not always correlate.

Although no adverse events were identified during the study period, this finding should not be interpreted as evidence of zero risk. Given the sample size and retrospective design, rare adverse events may not have been observed. Larger studies are needed to further characterize the safety profile. Lastly, procedural sedation success was determined from physician documentation, indicating successful completion of the procedure. Rescue medication use was also assessed through medication administration records, with any additional sedatives or analgesics recorded. These factors should be considered when interpreting the observed outcomes, as the retrospective design and reliance on clinical documentation may limit the precision of outcome assessment. In conclusion, this study observed frequent utilization of ketamine in the ED in KSA, particularly for procedural sedation and acute pain management. Ketamine was most commonly delivered as a combination therapy, with ketofol being the most common combination therapy delivered. In this study we found that no adverse events or procedural failures were documented.

## Figures and Tables

**Table 1:** Demographic and clinical characteristics of patients receiving ketamine (N = 192).

Variable	N = 192
**Age, years**	37 ± 15.35 33 (18–81)
**Gender** Male Female	131 (68.2) 61 (31.8)
**BMI, kg/m^2^**	26.03 ± 5.10 25.77 (Range, 14–39.44)
**Total ketamine dose (mg/kg)**	0.79 ± 0.55 0.65 (Range, 0.13–4)
**Total ketamine dose (mg)**	54.93 ± 35.81 50 (Range, 20–240)
**Ketamine Indication** Procedural sedation Agitation Pain RSI Status asthmaticus Status epilepticus	152 (79.2) 4 (2.1) 24 (12.5) 9 (4.7) 2 (1) 1 (0.5)
**Ketamine Use** Monotherapy Combined	31 (16.2) 161 (83.9)

**Abbreviations**: BMI, body mass index; RSI, rapid sequence intubation. Data are presented as mean ± SD or median (range) for continuous variables and n (%) for categorical variables. Ketamine dose is reported as mg/kg of body weight and total dose (mg).

**Table 2:** Ketamine dosing for procedural sedation.

Procedural Sedation Procedures	N = 152 N (%)	Ketamine Monotherapy N= 16			Ketamine Combination N = 136		
		Mean (mg/kg) Dose	Median Dose (mg/kg)	Range (Lowest–Highest)	Mean (mg/kg) Dose	Median Dose (mg/kg)	Range (Lowest–Highest)
Orthopedic reductions	129 (84.86)	0.82	0.69	0.25- 2.05	0.73	0.59	0.13–2.63
Incision and drainage procedures	6 (3.94)	1.76	1.76	1.76	0.81	0.62	0.49–1.32
Thoracic procedures (chest tube insertion)	4 (2.63)	1.08	1.08	0.63–1.53	1.05	1.05	0.95–1.14
Minor wound and soft tissue procedures	3 (1.97)	0.80	0.80	0.80	0.69	0.69	0.69
Neurological procedures (lumbar puncture)	2 (1.32)	0.69	0.69	0.45–0.93	0.70	0.70	0.46–0.93
Not specified	8 (5.20)	0.32	0.32	0.32	1.04	0.94	0.45–2.82

Data are presented as n (%) unless otherwise indicated. Dose is expressed as mg/kg. Range is presented as lowest–highest value. Not specified refers to procedures for which the indication was not documented in the electronic medical record.

**Table 3:** Ketamine dosing for procedural sedation (Ketofol).

Item		Type of Procedure					
		Orthopedic Reductions N = 129	Incision and Drainage Procedures N = 6	Thoracic Procedures (Chest Tube Insertion) N= 4	Minor Wound and Soft Tissue Procedures N = 3	Neurological Procedures (Lumbar Puncture) N = 2	Not Specified N= 8
Ketamine	Mean dose, mg/kg	0.74	1.05	1.06	0.75	0.70	1.17
	Median dose, mg/kg	0.64	0.97	1.05	0.75	0.70	1.03
	Dose Range, mg/kg	0.13–2.63	0.49–1.76	0.63–1.53	0.69–0.80	0.46–0.93	0.32–2.82
Propofol	Mean dose, mg/kg	0.65	1.69	1.58	–	1.67	1.04
	Median dose, mg/kg	0.57	0.62	1.58	–	1.67	0.94
	Dose Range, mg/kg	0.13–1.75	0.49–3.95	0.28–2.86	–	1.67	0.45–1.67

Data are presented as mean dose (mg/kg), median dose (mg/kg), and dose range (minimum–maximum). Ketamine and propofol doses are expressed as mg/kg of body weight. A dash (–) indicates that no patients received propofol for the corresponding procedure. Not specified refers to procedures for which the indication was not documented in the electronic medical record.

## Data Availability

The data are available from the corresponding author upon reasonable request. Data sharing is limited to de-identified data and is subject to institutional data-sharing policies and ethical requirements.
